# Laparoscopic management of giant abdominal cystic lymphangiomas: experience from the queen Fabiola children’s university hospital

**DOI:** 10.1186/s12893-025-03153-8

**Published:** 2025-10-03

**Authors:** Irene Nadine Kouna Tsala, Anna Poupalou, Helena Reusens, Gregory Rodesch, Eric Tobie Ntsobe, Eduardo Vieira Cardoso, Taisia Bollettini, Gianluca Gentilucci, Cyrille Leger Abega, Nasroola Damry, Basile Essola, Pierre Lingier

**Affiliations:** 1 Free University of Brussels, University Hospital of Brussels, Queen Fabiola Children’s University Hospital, Av. J.J. Crocq 15, Brussels, 1020 Belgium; 2 General Hospital of Garoua - Cameroon, Gannan Medical University, Ganzhou, China; 3 Department of Medical Sciences, Surgery and Neuroscience, Section of Pediatric Surgery, Policlinico Le Scotte, University of Siena, Viale Bracci 14, Sienna, 53100 Italy; 4 Department of Surgery, University of Douala, Douala, Cameroun

**Keywords:** Giant abdominal cystic lymphangioma, Minimally invasive surgery, Children

## Abstract

**Objective:**

Cystic lymphangioma is a benign malformities tumor of the lymphatic system. Its abdominal location is much rarer. We reviewed the cases of giant abdominal cystic lymphangioma managed laparoscopically. We used a video-assisted needle aspiration technique in our Pediatric Surgery Department of the Queen Fabiola Children’s University Hospital (HUDERF) in Brussels. From this study, we analyzed the clinical findings and surgical outcomes.

**Methods:**

We conducted a retrospective study in the Pediatric Surgery Department of HUDERF, Brussels. The study period was from January 1, 2014, to January 1, 2024. All patients (5) with a confirmed diagnosis of giant cystic lymphangiomas and who underwent laparoscopic surgery were included. Those who had been operated on by open surgery were excluded and those who had cyst lengths less than 5 cm (5). The laparoscopic management involved aspirating the cyst with a needle via a laparoscopic approach, followed by cyst resection either laparoscopically or through a video-assisted approach. Parameters that were studied include: age, sex, weight, symptoms, preoperative diagnosis, imaging assessment, localization, size of cyst, type of mass, surgical technique and duration, conversion to open surgery, morbidity, and histopathology. Data were analyzed using Microsoft Office Excel 2010 and SPSS.

**Results:**

We retrieved ten files of patients presenting with abdominal cystic lymphangioma. Two patients underwent exclusive open surgery and 3 patients with cystic lymphangiomas less than 5 cm in length were excluded. The mean age of the patients was 6 years with extremes ranging from 4 to 11 years. Male patients were predominant (3/5). The mean weight was 27.4 kg with extremes ranging from 17 to 55 kg. The most common symptom was abdominal pain. Preoperative diagnosis of abdominal cystic lymphangioma was made in 5 cases. Abdominal ultrasound was performed on all patients. Magnetic resonance imaging (MRI) was done in 3 patients, and Abdominal computed tomography (CT) was performed in 1 patient. The mass was multiloculated in 5 cases. Treatment consisted of pure laparoscopic resection of the cyst in 2 cases, and laparoscopic-assisted resection in 3 cases (with extra-peritoneal small bowel resection and mesenteric detorsion in 1 case). After a follow-up period of 5 years, the patients had a simple clinical course, and no recurrence was observed in our series.

**Conclusion:**

Giant abdominal cystic lymphangiomas can be managed laparoscopically using the video-assisted needle aspiration technique. This technique is highly effective and minimally invasive, regardless of cyst size.

## Introduction

Cystic lymphangiomas are dysembryopathies of the lymphatic system, responsible for a tumor-like syndrome due to lymphatic proliferation. It is a benign malformation tumor, most commonly affecting the cervicofacial region (75%) or the axilla (20%), and is typically diagnosed in childhood. Its abdominal localization is much rarer (5%) [1]. When located in the abdomen, it is generally found in the great omentum, mesentery, or retroperitoneum. Giant abdominal cystic lymphangiomas have a diameter ranging from 5 to 10 cm. Beyond 13 cm, they become symptomatic due to the compression of neighboring organs. They alter anatomical structures, become space-occupying lesions, and may affect vital functions, making the initial assessment even more challenging to perform before surgery [2]. In general, surgery for this type of lesion is challenging, and laparoscopic surgery is even more so, especially in pediatric patients.

Cystic abdominal lymphangiomas are increasingly managed using laparoscopic or video-assisted techniques, but giant lymphangiomas remain challenging for laparoscopic approaches. However, by using needle aspiration before dissection through laparoscopy, their management via laparoscopic surgery becomes more feasible.

We reviewed the cases of giant abdominal cystic lymphangioma in our Pediatric Surgery Department of the Queen Fabiola Children’s University Hospital (HUDERF) in Brussels. From this study, we analyzed the clinical findings and surgical outcomes.

## Methods

We retrospectively reviewed files of children who had been treated for abdominal cystic lymphangiomas at the Pediatric Surgery at HUDERF. Our study was submitted to and approved by the local ethics committee of HUDERF (CEH n°26/24). The requirement for consent to participate was waived. The study was conducted following the ethical principles of the Declaration of Helsinki and in compliance with the ICH Guideline for Good Clinical Practice.

The studied period was from January 1, 2014, to January 1, 2024. All patients with a confirmed diagnosis of cystic lymphangiomas and who underwent laparoscopic surgery were included in our study. Those who had been operated on by open surgery were excluded and those who had cyst length less than 5 cm were excluded. The radiological assessment of the mass included length. The operative technique involved either complete laparoscopic resection or laparoscopic assisted with extra-abdominal resection of the tumor. During the procedure, the patient was placed in a supine position with arms by their sides. A 5-mm optical trocar was inserted through an open technique at the umbilicus, and two additional 5-mm working trocars were placed on either side of the abdomen. The abdominal exploration was done first with a 30-degree optical camera. Aspiration of the mass was performed using an 18-gauge catheter, which was inserted through the skin at the site of the mass. The cyst was dissected after aspiration, using either a dissecting hook, Ligasure or a simple clamp, and extracted through the umbilical port. In cases the mass involved a bowel loop, an extra-corporeal anastomosis was performed.

Parameters that were studied included age, sex, weight, symptoms, preoperative diagnosis, imaging assessment, localization, size and type of mass, surgical technique, duration of the surgery, conversion to open surgery, morbidity, and histopathology. Data collection was done using the X-Care software and analyzed with Microsoft Office Excel 2010 and SPSS. All data generated or analysed during this study are included in this published article [and its supplementary information files].

## Results

During the study period, we included 10 patients who underwent surgery for abdominal cystic lymphangioma. Five patients who had been operated by open surgery were excluded and patients who had cyst length less than 5 cm were also excluded. The mean age of the patients was 6 years [4–11 years], with a predominance of male patients (3 boys, 2 girls). The mean weight at the surgery was 27,4 kg [17–55 Kg].

The most common symptom was abdominal pain in 3 cases, followed by intractable vomiting in 2 cases. Abdominal cystic lymphangioma was the preoperative diagnosis in 5 cases. Abdominal ultrasound was performed in all patients (5 cases). The combination of abdominal ultrasound and abdominal MRI was used in 3 cases, and an abdominal CT scan was performed in 1 case. The radiological assessment of the mass included length (Table 1). The mean length was 12,72 cm [7,3–17 cm]. During surgery, the mass was located in the mesentery in 4 patients and was multiloculated cystic. One case involved a mesogastric and pelvic location.

**Table 1:** Summary of Study Results

Patients	1	2	3	4	5
Age	5 years	11 years	5 years	4 years	5 years
Sex	M (Male)	F (Female)	M (Male)	F (Female)	M (Male)
Weight	17 kg	55 kg	28 kg	18 kg	19 kg
Symptoms	Abdominal pain	Abdominal pain	Intractable vomiting	Abdominal pain	vomiting
Preoperative Diagnosis	Abdominal cystic lymphangioma (ACL)	ACL	Small bowel volvulus	ACL	ACL
Imaging	Ultrasound (US) + MRI	US + MRI	US	US + CT + MRI	US + MRI
Localization	Mesentery	Mesentery	Mesentery	Mesentery	Mesenteric
Size	US: 17 × 4 × 10 cm MRI: 10 × 10 × 7 cm	US: NP MRI: 13 × 12 × 13 cm	US: 7,5 × 0,5 cm	US: NP CT: 133 × 106 × 54 mm	IRM13 × 9 × 7 cm
Mass Type	Multicystic	Multicystic	Multicystic	Multicystic	Multicystic
Surgical Procedure	Aspiration Resection + Anastomosis	Aspiration Excision of masses	Aspiration Detorsion + Resection + Anastomosis	Aspiration Excision of mass and extraction via umbilicus	Aspiration Excision of mass and extraction via umbilicus
Surgical Duration	4 h	1 h 30 min	NP	1 h 45 min	2 h
Conversion	+/-	No	+/-	No	+/-
Histopathology	ACL	ACL	ACL	ACL	ACL
Morbidity	-	-	-	-	-

*Legend*: US: Ultrasound; MRI: Magnetic Resonance Imaging; CT: Computed Tomography; ACL: abdominal cystic lymphangioma; NP: not precise

The surgical procedure performed on all patients involved laparoscopic excision of the mass, with cyst aspiration using a needle through the skin before excision. Treatment consisted of pure laparoscopic resection of the cyst in 2 cases, and laparoscopic-assisted resection of the cyst in 3 cases with extra-corporeal bowel resection and anastomosis was performed if necessary. One of them underwent mesenteric detorsion for volvulus. The mean duration of the procedure was 2H27 min [1H30–4 H] for the same surgeon (see table). The pathological examination of all the patient masses were in favor of an abdominal cystic lymphangioma, a multiloculated cystic mass with locules containing fibrin and lymphocytes. At a 5 years follow-up, the patients had a simple clinical course, with no recurrence observed in our series (Table 1).

## Discussion

In the study, we observed a predominance of male patients (3:2), with a mean age of 6 years (minimum 4 years and maximum 11 years). The age of patients in our series was lower than in some other studies [3–6]. We perform laparoscopic management in our patients, in contrast to other authors who prefer laparotomy, even with older patients [3–5]. Antao [2] and Lagausie [7] underwent laparoscopy in the patients at younger ages (as young as 2 years), suggesting that laparoscopic management of giant lymphangiomas can be successfully performed at any age, depending on the experience of the surgical team.

Abdominal pain is the most commonly reported symptom in 3 cases and vomiting is observed in 2 cases. The clinical presentation of abdominal cystic lymphangiomas is polymorphic, but abdominal pain remains the predominant symptom in clinical presentation [5]. Many authors [5, 6, 8] also report that abdominal pain may mimic other pathologies and can be associated with intestinal volvulus [2, 7, 8].

**Fig. 1:**
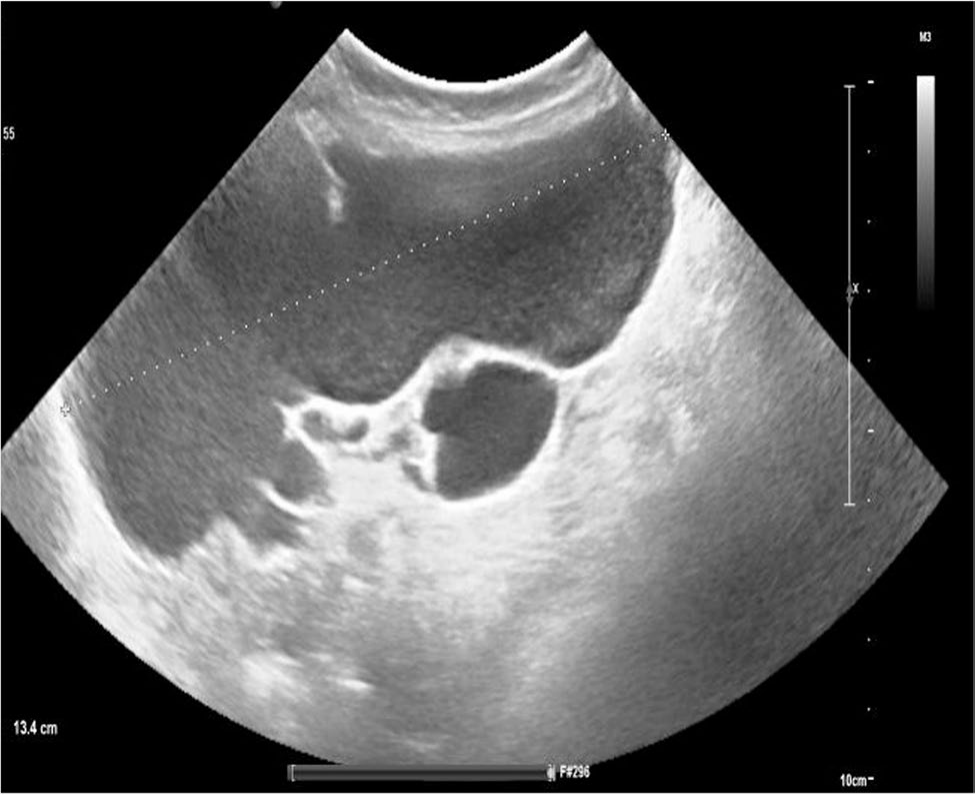
Ultrasonographic image

**Fig. 2:**
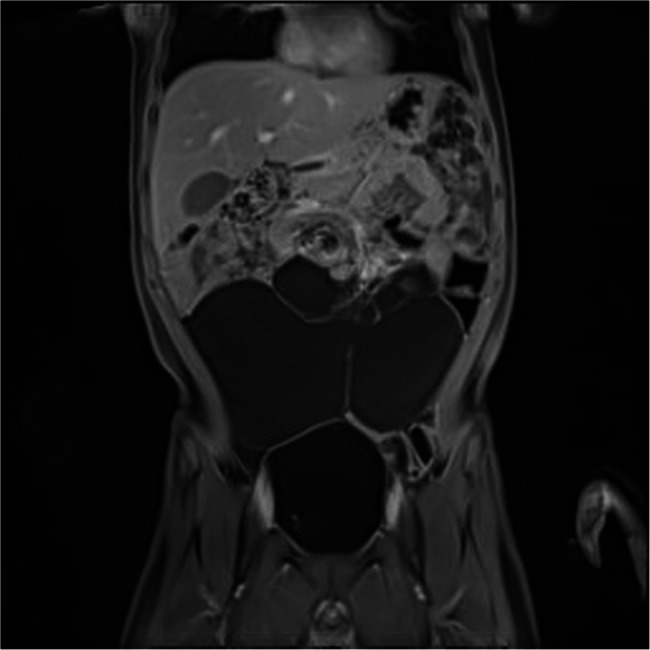
MRI image of abdominal lymphangioma

**Fig. 3:**
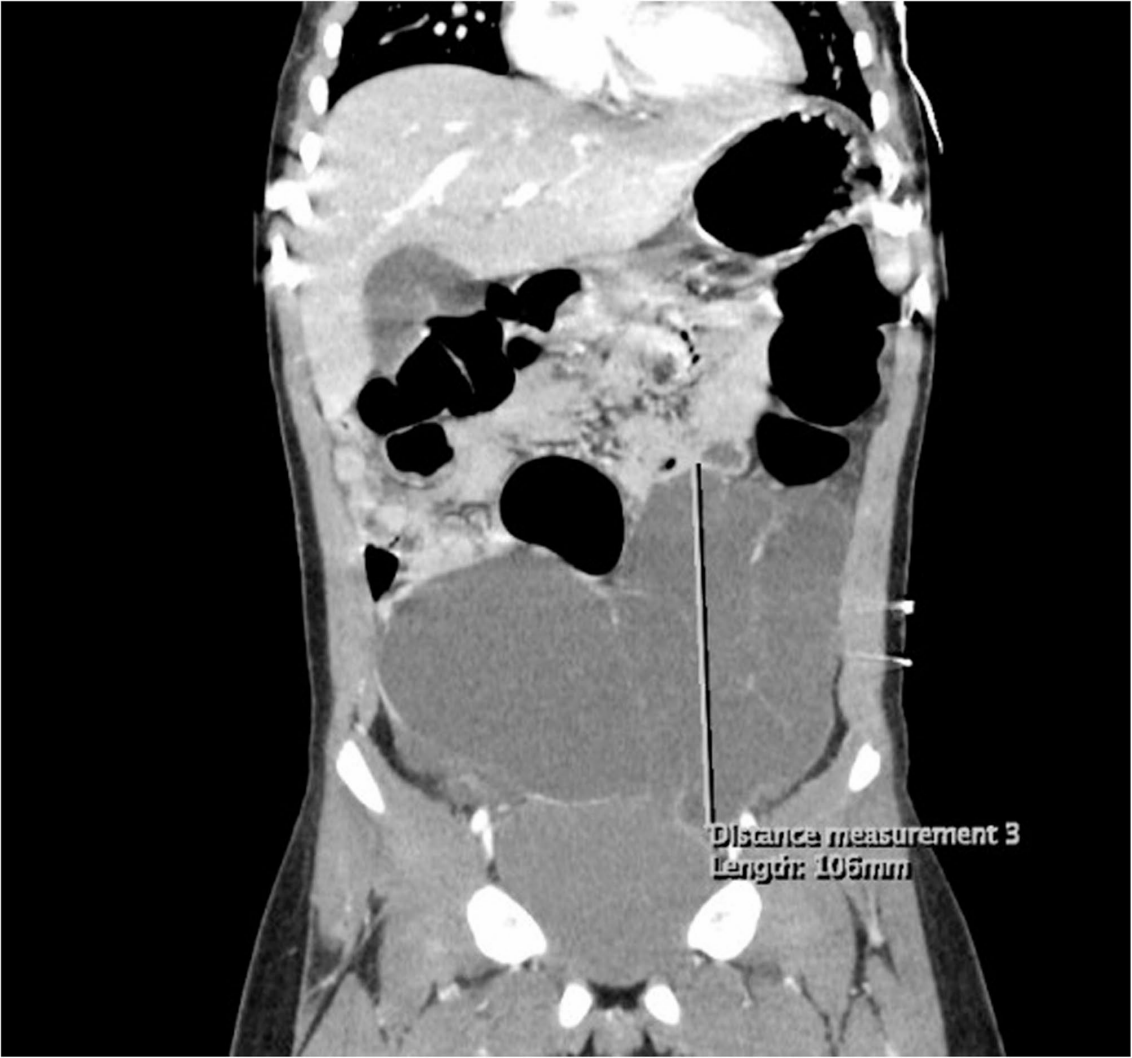
CT scan image of an abdominal cystic lymphangioma

The role of preoperative imaging is important in the management of abdominal cystic lymphangiomas. Ultrasound (Fig. 1), MRI (Fig. 2), and abdominal CT scans (Fig. 3) have provided accurate assessments of both the size, localization of the cyst, and its relationship with neighboring organs. These examinations are important for diagnosis and developing the patient management strategy, as emphasized by the other authors [5–8]. The combined use of MRI and ultrasound in three of our patients allows for a more detailed evaluation to understand the complexity of the cyst. Furthermore, these exams are essential to diagnose this condition, as noted in the pediatric case studies described by Rami [3] and Lagausie [7].

The average cyst size in our series was 12.72 cm, with extremes ranging from 7.5 cm to 17 cm. The mean age of the patients was 6 years. Xuping et al. [6] reported the case of a 14-year-old patient with a 7.8 cm cyst, treated laparoscopically, while Antao et al. [2] described the case of a 4-year-old patient with a 27 cm cyst, also treated laparoscopically. These results demonstrate that laparoscopic management of giant abdominal lymphangiomas is feasible, regardless of the cyst’s size. In contrast, other authors [3–5] opted for laparotomy in cases of giant cysts, citing the potential risk of incomplete resection with laparoscopic techniques.

**Fig. 4:**
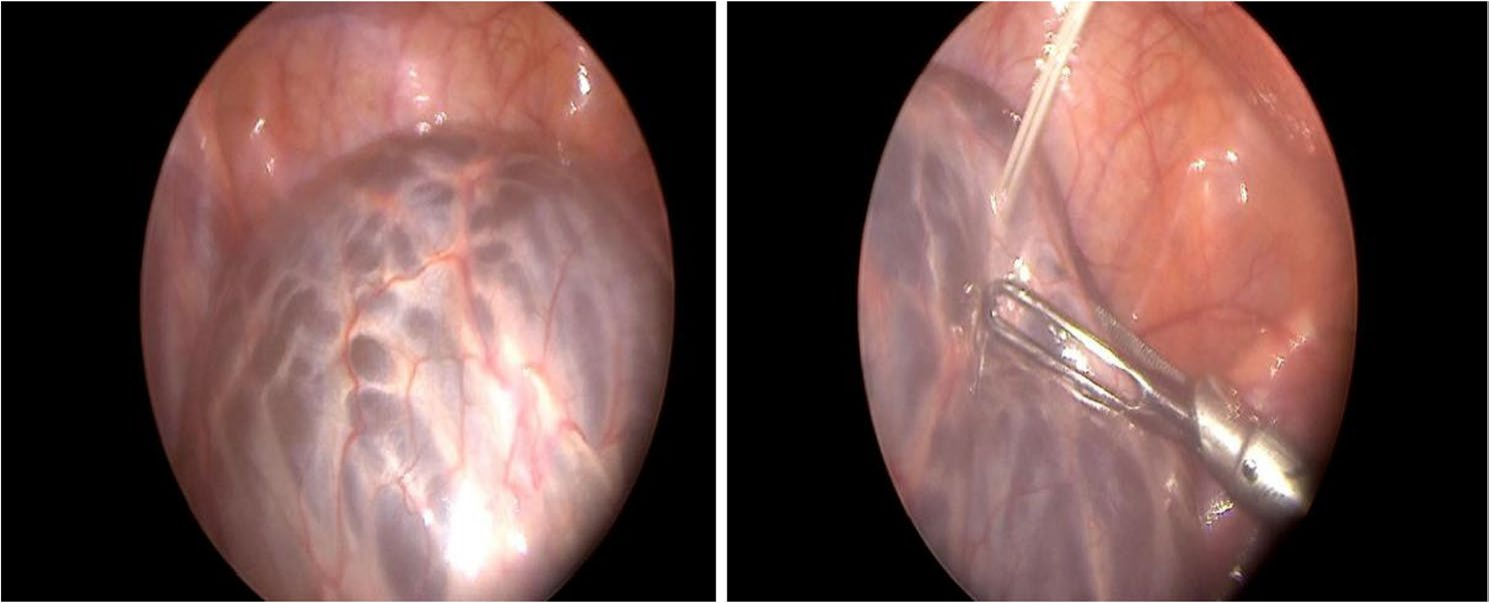
Drainage of the cyst and collection of fluid for analysis

**Fig. 5:**
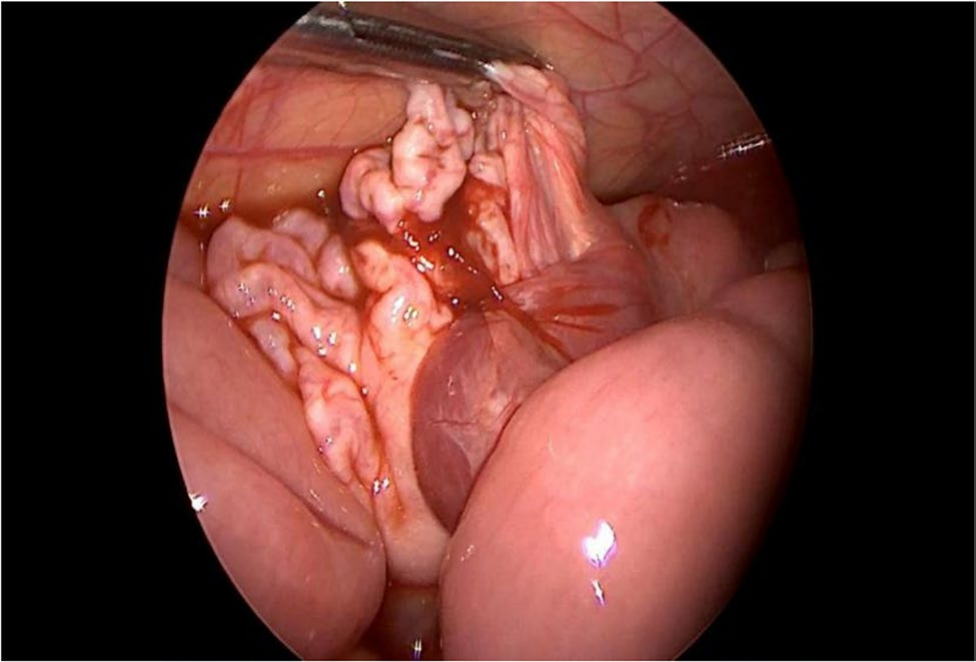
Resection of the cyst wall after drainage and extraction

After exploration, preoperative aspiration of the cystic lymphangioma before laparoscopic resection was a major step in our study (Fig. 4). The cyst is aspirated through the skin, the size is reduce and the resection will be simple (Fig. 5). This preoperative technique is useful for giant abdominal lymphangioma treatment in pediatric patients [9]. It reduces the cyst’s volume but also facilitates resection [2, 9]. Antao et al. [2] demonstrated that preoperative cyst aspiration can significantly reduce the risk of cyst rupture and lymphatic complications associated with dissection.

Spolianski et al. [10], in their work, noted that the first laparoscopic resection of retroperitoneal lymphangiomas was described in 1994 by Targona, who successfully performed the procedure on a 13 cm cyst in a 45-year-old adult. Although several authors have reported giant abdominal cystic lymphangiomas cases (Table N° 2), these cases were generally managed via laparotomy. Lagausie et al. [7] presented nine pediatric cases of abdominal cystic lymphangiomas managed laparoscopically, with uncomplicated postoperative outcomes. However, as the size of the cysts was not always specified in their study, it remains unclear whether these were classified as giant cysts. Very few studies address the laparoscopic management of giant lymphangiomas in pediatric cases. Antao et al. [2] reported a pediatric case of a giant abdominal cystic lymphangioma with 27 cm. They managed with a video-assisted needle aspiration technique followed by laparoscopic resection, with good results. Bertozzi et al. [11] presented 5 pediatric cases and 2 cases need needle aspiration before excision. This approach is less invasive than laparotomy for children.

**Table 2:** Comparative table with other authors

	Country	Years	Number of case	Age	Maximum length of the lymphangioma (cm)	Treatment	Morbidity
Our Study	Belgium	2024	5 cases	4–11 years	Case 1: 17 cm Case 2: 13 cm Case 3:13,3 cm Case 4:13 cm Case 5: 7,5 cm	Laparoscopy + percutateous aspiration	-
Rami Mohamed	Morroco	2012	3 cases	11 years 12 years 10 years	20 cm 20 cm 14 cm	laparotomy	-
Ahmad K. Abdulraheem	Jordania	2021	1 case	1 year 9 month	8 cm	laparotomy	-
Khalid Khattala	Morroco	2011	1 case	12 years	20 cm	laparotomy	-
Xuping Feng	China	2022	1 case	14 years	7,8 cm	laparoscopy	-
Nguyen Anh Tuan	Vietnam	2021	1 case	15 years	16 cm	laparotomy	-
Iuliana-Laura Candussi	Roumania	2024	1 case	2 years	23 cm	laparotomy	-
Antao and Coll	Island	2015	1 case	4 years	27 cm	laparoscopy	reccurence
Lagausie and Coll	France	2006	9 cases	5–14 years	Not precise	laparoscopy	-

A pure laparoscopic resection was performed in two cases, while three patients required laparoscopic-assisted resection. It combined with extra-corporeal bowel resection and anastomosis was performed if necessary. A mesenteric detorsion was done in one patient with volvulus. Giant abdominal cystic lymphangiomas with mesenteric volvulus, have been documented in the literature. Lagausie et al. [7] report similar cases where assisted laparoscopy played a major role in management. Several authors support laparoscopy as the best method for managing giant cystic lymphangiomas, due to its minimally invasive nature and their advantages [2, 7, 11].

The duration of the procedure was not specified in most of the series. In the study, it ranged from 1.5 h to 4 h, the average duration was 2.27 h. This variation reflects the complexity of certain procedures, which is related to several factors such as the size, localization of the masses, and unforeseen complications. The duration of surgery can also be affected sometimes by intestinal resection or volvulus detorsion. The average duration observed in our series is comparable to that of traditional laparotomy procedures, suggesting that laparoscopy should be considered a reliable and effective alternative. Surgeons would benefit from opting for laparoscopy, given the numerous advantages it offers to the patient compared to laparotomy.

The definitive diagnosis of cystic lymphangioma remains histological [1, 3, 8, 9], and in our study, all cases were confirmed by histopathological examination. After 5 years of follow-up, no recurrence was found in our study, a result similar to other studies [5, 6]. These studies demonstrate that complete laparoscopic resection leads to a low recurrence rate, while incomplete resection is associated with a 10–40% recurrence rate [5, 6, 11]. In case of recurrence, sclerotherapy using sclerosing agents can be used. New molecules are being highlighted in the management of lymphangiomas, particularly sirolimus. According to some authors [11, 12], it is effective in cases of multiloculated cystic lymphangiomas or in cases of incomplete surgical resection of the cyst.

## Conclusion

Giant abdominal cystic lymphangiomas are a challenge for laparoscopic approaches. Cyst aspiration with a needle before laparoscopic dissection is a minimally invasive approach, regardless of the cyst’s size. This technique can prove effective with good results. Long-term follow-up and studies with larger series will help refine this surgical strategy.

## Data Availability

All data generated or analysed during this study are included in this published article.

## Abbreviations

MRI: Magnetic Resonance Imaging
CT: Computed Tomography
Kg: Kilogram
Cm: Centimeter
HUDERF: Queen Fabiola Children’s University Hospital
